# Melatonin Rescues Photosynthesis and Triggers Antioxidant Defense Response in *Cucumis sativus* Plants Challenged by Low Temperature and High Humidity

**DOI:** 10.3389/fpls.2022.855900

**Published:** 2022-04-27

**Authors:** Bakht Amin, Muhammad Jawaad Atif, Huanwen Meng, Muhammad Ali, Shuju Li, Hesham F. Alharby, Ali Majrashi, Khalid Rehman Hakeem, Zhihui Cheng

**Affiliations:** ^1^College of Horticulture, Northwest A&F University, Yangling, China; ^2^Horticultural Research Institute, National Agricultural Research Centre, Islamabad, Pakistan; ^3^Tianjin Kerun Cucumber Research Institute, Tianjin, China; ^4^Department of Biological Sciences, Faculty of Science, King Abdulaziz University, Jeddah, Saudi Arabia; ^5^Princess Dr. Najla Bint Saud Al-Saud Center for Excellence Research in Biotechnology, King Abdulaziz University, Jeddah, Saudi Arabia; ^6^Department of Biology, College of Science, Taif University, Taif, Saudi Arabia; ^7^Department of Public Health, Daffodil International University, Dhaka, Bangladesh

**Keywords:** antioxidant enzyme, cucumber, high-humidity, low-temperature, melatonin

## Abstract

Environmental factors such as low temperature (LT) and high humidity (HH) hinder plant growth and development in plastic tunnels and solar greenhouses in the cold season. In this study, we examined the effect of melatonin (MT) on shoot-based tolerance to LT and HH conditions in cucumber (*Cucumis sativus*) seedlings and explored its underlying mechanism. LT and HH stress inhibited growth and biomass accumulation, produced leaf chlorosis, led to oxidative stress, lowered chlorophyll and carotenoid contents, reduced photosynthetic and photosystem II (PSII) activities, and increased the level of intercellular carbon dioxide and the non-photochemical quenching of photosystem I (PSI) and PSII. However, foliar application of MT significantly improved the morphological indices and photosynthetic efficiency of cucumber seedlings, which entailed the elevation of electrolyte leakage, lipid peroxidation, and reactive oxygen species accumulation by boosting the antioxidant enzyme defense system under LT and HH conditions. Additionally, the measurement of nitrogen (N), magnesium (Mg), and iron (Fe) contents in roots and leaves showed that MT significantly augmented the nutrient uptake of cucumber seedlings exposed to LT and HH stresses. Furthermore, MT application increased the transcripts levels of genes encoding antioxidant enzymes under LT and HH conditions, whereas treatment with LT and HH suppressed these genes, suggesting that MT application increases the LT and HH tolerance of cucumber seedlings. Overall, our results suggest that MT application increases the tolerance of cucumber seedlings to LT and HH stress by enhancing the plant morphometric parameters, regulating PSI and PSII, and activating the antioxidant defense mechanism. Thus, the exogenous application of MT could be potentially employed as a strategy to improve the LT and HH tolerance of cucumber.

## Introduction

The growth and productivity of plants are negatively impacted by numerous abiotic stresses, which occur throughout the plant life cycle in the natural environment. Tropical plants are susceptible to environmental factors such as low temperature (LT) (Zhao et al., 2017) and high humidity (HH) (Han et al., 2019), which hamper plant growth and impose physiological stress. Any compromise of plant growth parameters can eventually lead to food scarcity, as has been observed recently in many plant species in response to global climatic changes (Han et al., 2019; Zhang et al., 2021). Weather-related changes and frequent fluctuations in temperature have been shown to affect the growth, development, and biochemical and physiological attributes of crop plants (Sakina et al., 2019).

Photosynthesis is the main physiological process affected by chilling stress. Physiological changes observed in plants under cold conditions are mainly the result of a disturbance in biochemical reactions operating in the photosynthetic machinery, which eventually impedes plant growth. Cold stress leads to growth modifications that limit energy usage and inhibit photosynthesis (Ruelland and Zachowski, 2010). Previous studies reported that an increase in temperature and humidity impairs photosynthesis by reducing turgor and limiting carbon dioxide (CO_2)_ availability because of stomatal closure and damage to photosystem I (PSI) and photosystem II (PSII) components (Yang et al., 2012; Han et al., 2019). Stressed plants have been shown to accumulate reactive oxygen species (ROS) such as hydrogen peroxide (H_2_O_2_) and superoxide anion (O_2_^–^) in the cellular and subcellular compartments, which damages cells, leading to programmed cell death (PCD) and a reduction in plant growth and development (Marta et al., 2016; Raja et al., 2017). To escape the detrimental effects of oxidative stress caused by unfavorable abiotic conditions, plants have evolved several mechanisms, one of which involves an increase in the endogenous levels of antioxidant enzymes such as superoxide dismutase (SOD), catalase (CAT), peroxidase (POD), ascorbate POD (APX), glutathione reductase (GR), monodehydroascorbate reductase (MDHAR), and dehydroascorbate reductase (DHAR) (Gupta et al., 2016; Mehla et al., 2017; Hasanuzzaman et al., 2020).

Numerous studies have shown that low temperature (LT) stress impairs plant nutrient uptake and accumulation, photosynthesis, and the antioxidant defense system (Anwar et al., 2018; Ciura and Kruk, 2018). The most innovative strategy for protecting plants against individual and concurrent abiotic stresses is to use plant bio stimulators or hormones that can help the plant to adapt to and evade the stress (Eremina et al., 2016; Li et al., 2016). Plants detect cooling signals *via* hormones, which cause biochemical and physiological changes. Melatonin (MT) (N-acetyl-5-methoxytryptamine) is a recent addition to the list of phytohormones in angiosperms. MT is a small molecule first discovered in the bovine pineal gland (Lerner et al., 1958). This discovery led to the investigation of the impacts of MT on animal biology, including physiological processes such as circadian rhythm, sleep, body temperature, physical activity, retinal physiology, sexual activity, seasonal reproduction, and the immune system (Zhang et al., 2021). Recently, Erdal (2019) revealed that MT plays a critical role in plants as well. MT has been found to stimulate vegetative growth in plants by promoting the formation of lateral roots and increasing the shoot length and leaf area. MT has also been reported to function as a signaling molecule that protects plants from adverse environmental conditions (Shi et al., 2015; Li et al., 2016; Arora and Bhatla, 2017). Lei and colleagues used MT in carrot suspension cells to reverse the damage caused by freezing stress (Lei et al., 2004). Furthermore, MT has been found to enhance cucumber (*Cucumis sativus* L.) seed germination by preventing the peroxidation of membrane lipids during cold stress (Posmyk et al., 2009). Zhao et al. (2016) and Anwar et al. (2018) found that MT and 5-aminolevulinic acid (ALA) improve antioxidant defense, regulate photosynthesis and nutrient uptake, and accelerate ROS scavenging in cucumber plants exposed to cold stress. Although cucumber is an all-season salad crop with huge commercial importance, the impact of LT and high humidity (HH) stress on this important plant species has not been carried out in a systematic way.

Cucumber (Cucurbitaceae) is a well-known vegetable crop grown all over the world. Today, cucumber is cultivated commercially in all seasons in fields and nurseries in different agroclimatic zones throughout the year. Cucumber plants require adequate water and moderate temperature for proper growth. Although many new cucumber cultivars have been developed for different seasons, LT has a negative impact on the growth and productivity of all cultivars (Lee et al., 2002). A previous study demonstrated that cold stress causes physiological injury to cucumber seedlings, particularly when grown in plastic tunnels and under greenhouse conditions during the winter season. Cucumber plants grown under such conditions produce small fruits with low yields (Cabrera and Saltveit, 1990). Although it has been demonstrated that MT is critical for antioxidant defense and photosynthesis in cucumber seedlings exposed to LT, the optimal dose of MT and its impact on LT and HH tolerance have not been thoroughly investigated. In this study, we investigated the impact of exogenous MT application on the morphophysiological characteristics of cucumber plants and on the antioxidant defense mechanisms under LT and HH stress. We observed that MT could improve LT and HH tolerance, and found that MT applied at a concentration of 200 μm was the most effective. To the best of our knowledge, this is the first report on the effect of MT on the LT and HH tolerance of cucumber plants and the underlying mechanisms.

## Materials and Methods

### Plant Materials and Growth Conditions

Cucumber (*Cucumis sativus* L.) cultivar Zhongnong 37 was used in this study, in the controlled growth chamber at College of Horticulture Northwest A&F University, China. The seeds were soaked first in hot water at 55^°^C for 15 min and then in room-temperature water for 6–8 h, before being placed in an incubator at 25 ± 1^°^C for sprouting. Following germination on moist filter paper in Petri dishes, the sprouted seeds were shifted to a 32-hole plug tray filled with nutrient medium, and incubated under controlled conditions: 25^°^C/18^°^C day/night temperature, 80% relative humidity, 12-h light/12-h dark photoperiod, and 2,000 Lx photosynthetically active radiation. The seedlings were routinely managed. When the cotyledons were fully opened, uniform-sized seedlings were transferred to plastic pots. Cucumber seedlings at the fully developed 2-leaf stage were divided into two groups; 50% of the seedlings were sprayed with 200 μm MT once a day at 8 p.m. for 5 days, and the remaining 50% were sprayed with distilled water at the same frequency. The optimum concentration of MT was selected based on a preliminary experiment (unpublished data). Both the water and MT treated groups were divided into two sets. One set of plants was placed in a growth chamber under LT and HH (LTHH) stress conditions (15/10^°^C day/night temperature and 95% humidity), whereas the other set was placed under control conditions (25/18^°^C day/night temperature and 80% humidity). The experiment was repeated three times (*n* = 3), and each replication comprised 20 plants. Thus, four treatments were conducted overall: (i) Control, seedlings incubated under control conditions; (ii) Control + MT, seedlings subjected to control conditions and then sprayed with MT; (iii) LTHH, seedlings subjected to LT and HH; (iv) LTHH + MT, seedlings subjected to LT and HH conditions and then sprayed with MT.

### Sampling

The morphological parameters of cucumber seedlings were measured 6 days after treatment (DAT). Leaves were sampled at 0, 2, 4, and 6 DAT to monitor dynamic changes. Photosynthetic parameters were evaluated in intact leaves.

### Morphometric Studies

The plant height and root length were measured using a scale, while the shoot fresh weight (FW), shoot dry weight (DW), and total DW were determined using a digital balance (Mettler Toledo, model PL303, United States). The strong seedling index (SSI) was calculated by the following equation:


$$S\,t\,r\,o\,n\,g\,S\,e\,e\,d\,l\,i\,n\,g\,I\,n\,d\,e\,x=\,\left(\frac{S\,t\,e\,m\,d\,i\,a\,m\,e\,t\,e\,r}{P\,l\,a\,n\,t\,h\,e\,i\,g\,h\,t}+\frac{R\,o\,o\,t\,D\,W}{S\,h\,o\,o\,t\,D\,W}\right)\times T\,o\,t\,a\,l\,D\,W$$

S⁢t⁢r⁢o⁢n⁢g⁢S⁢e⁢e⁢d⁢l⁢i⁢n⁢g⁢I⁢n⁢d⁢e⁢x

=

$$\,\left(\frac{S\,t\,e\,m\,d\,i\,a\,m\,e\,t\,e\,r}{P\,l\,a\,n\,t\,h\,e\,i\,g\,h\,t}+\frac{R\,o\,o\,t\,D\,W}{S\,h\,o\,o\,t\,D\,W}\right)$$

×T⁢o⁢t⁢a⁢l⁢D⁢W

### Leaf Photosynthetic Gas Exchange Parameters

Gas exchange parameters including, photosynthesis (pn), stomatal conductance (Gs), intercellular CO_2_ concentration (Ci), and transpiration rate (E) were measured using a portable photosynthesis system (LI-COR 6800XT, Lincoln, NE, United States). To measure these parameters, seedlings of the same size and with intact leaves were chosen from each treatment and maintained under control conditions for 1 h (from 11 a.m. to 12 p.m.) (Anwar et al., 2018).

### Chlorophyll Fluorescence Parameters

The chlorophyll fluorescence indices were measured using multispectral fluorescence imaging technology (FC800 FluorCam, PSI Czech). To measure the maximum quantum yield (F_v_/F_m_), non-photochemical quenching (NPQ), photochemical quenching (qP), and electron transport rate (ETR), plants were dark-adapted for 30 min post-treatment. Maximum fluorescence was recorded using a 0.8-s pulsed light at 4,000 μmol s^–1^ m^–2^. F_v_/F_m_ was determined for each plant using a whole leaf as the area of interest (Zhang et al., 2020).

### Preparation of Enzyme Extract

Leaf samples (0.5 g FW) were homogenized at 4°C in 5 ml of pre-cooled 50 mm potassium phosphate buffer (pH 7.8) containing 0.2 mM EDTA-Na_2_, and 1% (w/w) insoluble polyvinylpyrrolidone (PVP). The homogenate was centrifuged at 15,000 × *g* for 20 min at 4°C, and the supernatant was used for measuring antioxidant enzyme activity and malondialdehyde (MDA) content.

### Antioxidant Enzyme Activity Assay

To conduct antioxidant enzyme activity assays, samples were prepared as described previously (Amin et al., 2021). To measure the SOD (EC 1.15.1.1) activity, nitro blue tetrazolium (NBT) was added to the enzyme extract and exposed to fluorescent light for 20 min, after 20 min, the absorbance of the reaction mixture was measured using a spectrophotometer at 560 nm (Stewart and Bewley, 1980). The activity of POD (EC 1.11.1.7) was measured as described previously (Polle et al., 1994). Briefly, a 20-μl aliquot of the enzyme extract was mixed with 3 ml of the POD reaction mixture (0.1 mm phosphate buffer [pH 6.0], 16 mm guaiacol, and 19 μl of 10% (w/v) H_2_O_2_), and POD activity was recorded at 30-s intervals for 3 min at a wavelength of 470 nm. The activity of CAT (EC 1.11.1.6) was measured *via* H_2_O_2_ reduction, as described previously (Chance and Maehly, 1955). Briefly, a 50-μl aliquot of enzyme extract was mixed with 2.5 ml of the reaction mixture [0.1 mM phosphate buffer (pH 7.0) and 0.1 mm H_2_O_2_], and absorbance was measured at 240 nm. The activity of APX was determined by adding 0.2 ml of enzyme extract to the reaction buffer containing 50 mm phosphate buffer (pH 7.0), 0.1 mm EDTA, 0.5 mm AsA, and 1.0 mm H_2_O_2_, followed by measuring the absorbance with a spectrophotometer at 290 nm (Nakano and Asada, 1981). GR activity was measured according to the method of Rao et al. (1996). Briefly, 1 ml of reaction buffer (100 mM phosphate buffer, 0.5 mM GSSG, 2 mm EDTA, and 0.2 mM nicotinamide adenine dinucleotide phosphate (NADPH) was added to 0.25 ml of enzyme extract, shaken thoroughly, and incubated at 100°C for 10 min. Activities of MDHAR and DHAR were assayed using the protocol of Ma and Cheng (2004). To assay the MDHAR activity, the reaction buffer containing 925 μl of 50 mm 1-piperazineethanesulfonic acid (HEPES) phosphate buffer (pH 7.6), 10 μl of 25 mm NADPH, and 10 μl of 250 mM AsA was mixed with 1 ml of enzyme solution, and absorbance was noted at 340 nm. To determine DHAR activity, 20 μl of enzyme extract was added to a mixture containing 50 μl of 4 mm DHA and 25 μl of 100 mm GSH. Then, 905 μl of 0.1 M phosphate buffer (pH 7) was added to the reaction mixture, and absorbance was recorded at 265 nm.

### Determination of Chlorophyll and Carotenoid Content

According to Shah et al. (2019) leaf pigments were estimated by acetone under dim light. A 0.25 g leaf sample was ground by using TissueLyzer, then added 5 ml of 80% acetone before centrifugation at 10,000 × *g* for 5 min. After centrifugation, the supernatant was collected, and the absorbance for chlorophyll and carotenoid contents were noted at 663.6 and 646.6 nm wavelength, respectively.

### Determination of Malondialdehyde Content and Electrolyte Leakage

The MDA content was measured as described previously (Tiwari et al., 2010). Briefly, 2 ml of thiobarbituric acid (TBA) and 1 ml of enzyme extract were mixed with 5% TCA (v/v), and the mixture was heated in a water bath for 15 min. Subsequently, the reaction mixture was incubated at room temperature for a while, and absorbance was measured through a spectrophotometer at 450, 532, and 600 nm. A standard curve was used to calculate the MDA content.

To detect electrolyte leakage (EL), 20 leaf discs were immersed in test tubes containing deionized water. Electrical conductivity (EC) was measured before and after heating the samples in a water bath at 50°C for 25 min. Then, the tubes were heated again at 100°C for 10 min, and electrolyte leakage (%) was used determined using the following equation (Jan et al., 2018):


$$E\,l\,e\,c\,t\,r\,o\,l\,y\,t\,e\,l\,e\,a\,k\,a\,g\,e=\frac{E\,C_{b}-E\,C_{a}}{E\,C_{c}}\times 100$$

where EC_a_, EC_b_, and EC_c_ represent the EC of samples before heating, heated at 25°C, and heated at 100°C, respectively.

### Measurement of O_2_^–^ and H_2_O_2_ Contents

The contents of O_2_^–^ and H_2_O_2_ were measured as described previously (Zhang et al., 2010). To determine the O_2_− concentration, a 0.2-g sample was ground in phosphate buffer (pH 7.8) and centrifuged at 10,000 × *g* for 10 min. The reaction mixture contained 75 μl phosphate buffer (pH 7.8), and 25 μl 10 mm was mixed with the upper phase, and incubated for 20 min. Subsequently, 100 μl of the supernatant was mixed with 1 ml of 7 mm α-naphthalene diamine hydrochloride, 1 ml of 17 mM sulphanilamide (in 30% acetic acid), and 3 ml of ether, and centrifuged at 5,000 × *g* for 10 min. After centrifugation, the absorbance of each sample was measured at 240 nm, and the O_2_^–^ content was calculated using a standard curve.

To determine the H_2_O_2_ content, a 0.5-g sample was ground in 5 ml of acetone, and the sample was centrifuged at 5,000 × *g* for 5 min at 4°C. Then, 200 μl of ammonia solution and 100 μl of titanium tetrachloride were added to 1 ml of supernatant. The mixture was centrifuged at 12,000 × *g* for 10 min, and absorbance was measured at 410 nm.

### Determination of Nutrient Contents

The leaves and roots of the cucumber seedlings were separately dried at 65^°^C for 2 days and then milled into powder using a TissueLyser. A microwave digestion system (MARS6; CEM, United States) was used to digest 0.1–0.2 g of the powdered tissue with 4 ml of nitric acid and 1 ml of H_2_O_2_. The digested sample was filtered through a 0.45-μm Millipore filter, and stored in plastic vials for further analysis. An Induced Couple Plasma Mass Spectrometer (ICP-MS, NexION 300X; PerkinElmer: NexION 300X, NY, United States) was used to determine the iron (Fe) and magnesium (Mg) contents (Anwar et al., 2018). The total nitrogen (N) content of the root and leaf tissues was measured using the Kjeldahl method.

### RNA Extraction and Gene Expression Analysis

Total RNA was extracted from cucumber leaf samples using an RNA Isolation Kit (Omega Bio-Tek, Doraville, GA, United States) according to the manufacturer’s instructions. The quality of the RNA samples was assessed using a NanoDrop spectrometer: (1000 spectrophotometer NanoDrop Technologies, Wilmington, DE, United States). First-strand complementary DNA (cDNA) was synthesized from 1 μg total RNA using a PrimeScript RT Reagent Kit with a gDNA Eraser (Takara Bio, Shiga, Japan). The resulting cDNA was quantified and diluted to 200 μg. To analyze gene expression, primers were designed using Primer3web (version 4.1) (Supplementary Table 1), and real-time quantitative PCR (RT-qPCR) was performed on the Bio-Rad CFX 134 Connect Real-Time PCR Detection System using a SYBR Green qPCR Kit: TaKaRa, United States. A cucumber *ACTIN* gene was used for data normalization. Three technical replications were performed, and relative gene expression was calculated using the 2^–ΔΔCt^ method (Zhang et al., 2021).

### Statistical Analysis

The data were presented as mean ± SD, and statistically analyzed using Tukey’s multiple comparison test with GraphPad PRISM version 8.00: Two-way ANOVA followed by Tukey’s multiple comparisons test was performed using GraphPad Prism version 8.0.0 for Windows, GraphPad Software, San Diego, California, United States.^1^ Significant differences were indicated with asterisks, as follows: *P < 0.05, ^**^P < 0.01, ^***^P < 0.001, ^****^P < 0.0001. Differences with *P* > 0.05 were considered non-significant (ns).

## Results

### Morphometric Study

In the present study, we examined the effect of MT dose on the LT and HH tolerance of cucumber seedlings. At 6 DAT, we measured the seedling height, total FW, total DW, shoot FW and DW, root length, and SSI. The LTHH treatment significantly reduced seedling height, shoot FW, shoot DW, root length, SSI, and total DW by 22.05, 23.86, 24.26, 35, 25.48, and 27.98%, respectively, compared with seedlings grown under normal (control) conditions. On the other hand, pretreatment with MT (LTHH + MT) significantly ameliorated the damaging effect of LTHH conditions and increased seedling height, shoot FW, shoot DW, root length, SSI, and total DW by 11.27, 11.16, 16.74, 20, 17.19, and 20.50%, respectively, compared with control plants (Table 1).

**TABLE 1:** Effect of melatonin on cucumber seedling morphology under low-temperature and high-humidity

Treatment	Plant height (cm)	Shoot fresh weight (g plant^–1^)	Shoot dry weight (g plant^–1^)	Root length (cm)	SSI	Total dry weight (g plant^–1^)
Control	6.80 ± 0.2a	10.93 ± 0.04a	0.86 ± 0.07b	26.66 ± 1.52a	0.83 ± 0.02a	1.02 ± 0.05a
Control + MT	6.93 ± 0.15a	11.56 ± 0.75a	0.89 ± 0.09a	26.66 ± 2.51a	0.83 ± 0.02a	1.05 ± 0.07b
LTHH	5.30 ± 0.10c	8.32 ± 0.04c	0.65 ± 0.04d	17.33 ± 0.57c	0.62 ± 0.01c	0.73 ± 0.04d
LTHH + MT	6.03 ± 0.15b	9.71 ± 0.32b	0.72 ± 0.09c	21.33 ± 1.57b	0.69 ± 0.02b	0.81 ± 0.06c

*Control (25/18°C); Control + MT (200 μM melatonin, 25/18°C); LTHH (15/10°C, 95%); LTHH + MT (200 μM melatonin, 15/10°C, 95%). SSI (strong seedling index). Same letters represent no significant differences, while different letters represent significant differences among treatments (P > 0.05 was non-significant). Data are means ± SD of three replicates.*

### Leaf Photosynthetic Gas Exchange

**FIGURE 1:**
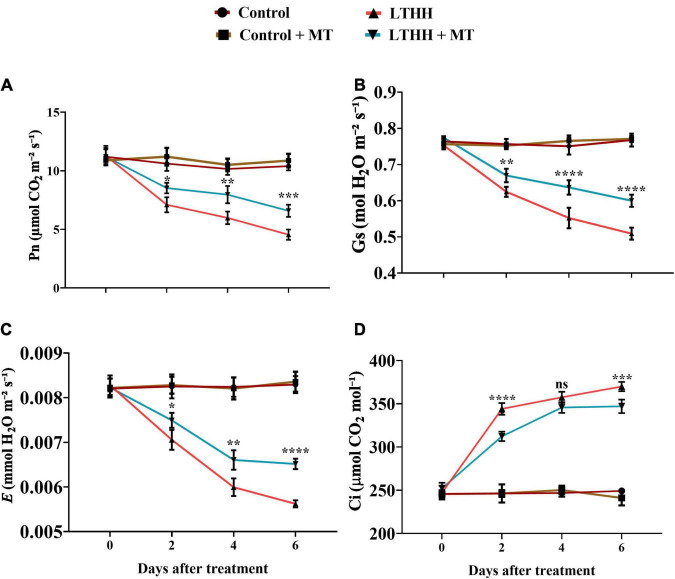
Effect of exogenous melatonin on net photosynthesis **(A)**, stomatal conductance **(B)**, transpiration rate **(C)**, and intercellular concentration of carbon dioxide rate **(D)** of cucumber seedling under low-temperature and high-humidity stress. Control (25/18°C); Control + MT (200 μM melatonin, 25/18°C); LTHH (15/10°C, 95%); LTHH + MT (200 μM melatonin, 15/10°C, 95%). Results were presented as means ± SD (*n* = 3), significance values were presented as: *P < 0.05; **P < 0.01; ***P < 0.001; ****P < 0.0001; while non-significant (ns) (*P* > 0.05).

Next, we monitored the gas exchange parameters. Plants treated with or without MT showed no significant changes in gas exchange parameters under control conditions (Figure 1). However, in the LTHH treatment, the values of Pn, Gs, and E decreased significantly, and the decline was more prominent in the later stages than in the early stages of growth. Compared with the control plants, the values of Pn, Gs, and E in LTHH treated plants decreased by 59.39, 33.35, and 31.47%, respectively, at 6 DAT, while that of Ci increased by 51.51%. However, exogenous MT application significantly mitigated this decline; at 6 DAT, values of Pn, Gs, and E, were increased by 37.93, 20.69, and 21%, respectively, in the LTHH + MT treatment compared with the water pretreatment, while that of Ci decreased by 40.94% (Figure 1).

### Chlorophyll Fluorescence

**FIGURE 2:**
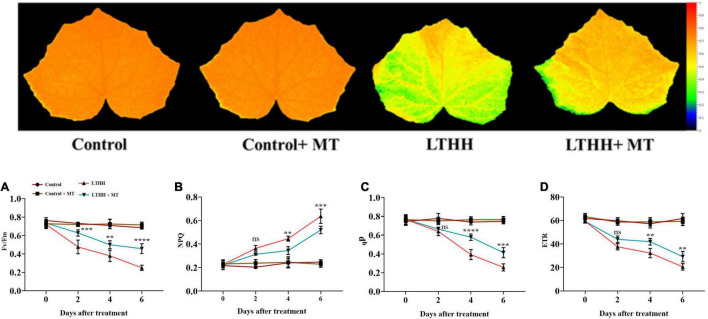
Effect of exogenous melatonin on chlorophyll fluorescence parameters PSII maximum efficiency **(A)**, non-photochemical quenching **(B)**, photochemical quenching **(E)**, and electron transport rate **(D)** of cucumber seedling under low-temperature and high-humidity stress. Control (25/18°C); Control + MT (200 μM melatonin, 25/18°C); LTHH (15/10°C, 95%); LTHH + MT (200 μM melatonin, 15/10°C, 95%). Results were presented as means ± SD (*n* = 3), significance values were presented as: *P < 0.05; **P < 0.01; ***P < 0.001; ****P < 0.0001; while non-significant (ns) (*P* > 0.05).

Next, we sought to determine the chlorophyll fluorescence indices, including Fv/Fm, qP, NPQ, and ETR, in cucumber seedling leaves to elucidate whether MT can alleviate the effects of LT and HH stress on photosynthesis-related parameters (Figure 2). Under control conditions, no significant changes were observed between plants treated with and without MT. However, compared with the water pretreatment, the LTHH treatment significantly reduced Fv/Fm, qP, and ETR by 63.42, 65.71, and 66.73%, respectively, at 6 DAT, and increased the NPQ by 157.97% (Figure 2). The LTHH + MT treatment alleviated the photochemical reaction and balanced the PSII efficiency in cucumber plants. The LTHH + MT treatment significantly increased Fv/Fm, qP, and ETR, by 35.98, 46.01, and 50.75%, respectively, while that of NPQ was decreased by 127.11%, at 6 DAT compared with the control (Figures 2A–D).

### Antioxidant Enzyme Activity

**FIGURE 3:**
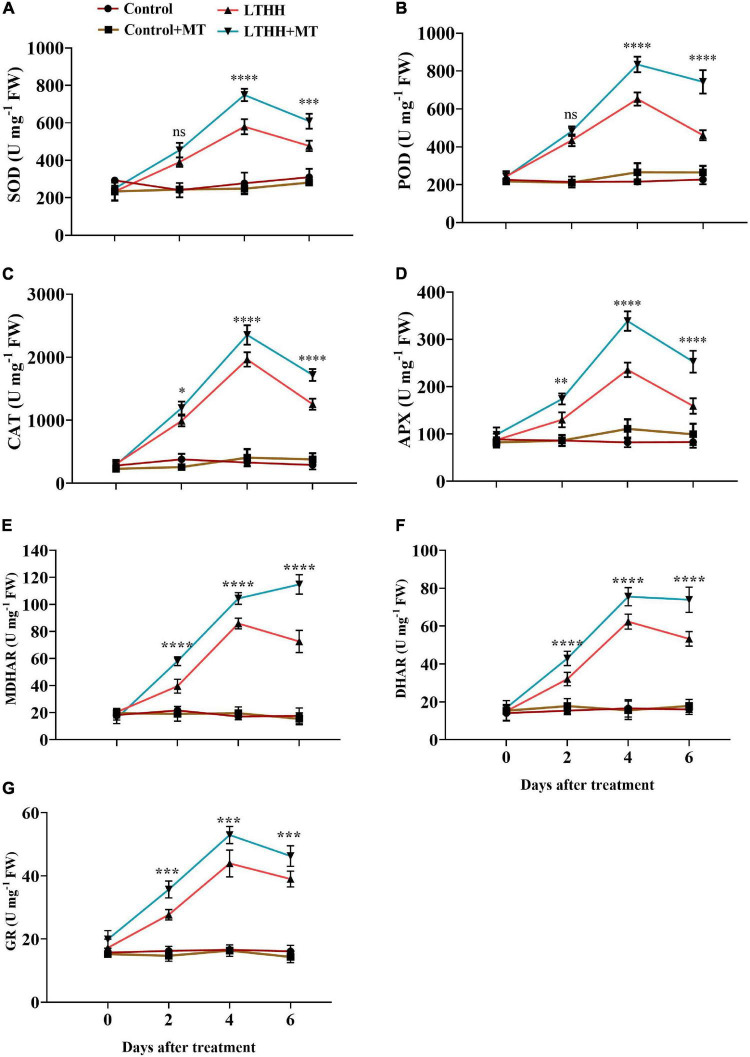
Effect of exogenous melatonin on superoxide dismutase **(A)**, peroxidase **(B)**, catalase **(C)**, ascorbate peroxidase **(D)** monodehydroascorbate reductase **(E)**, dehydroascorbate reductase **(F)**, and glutathione reductase **(G)**, activities of cucumber seedling under low-temperature and high-humidity stress. Control (25/18°C); Control + MT (200 μM melatonin, 25/18°C); LTHH (15/10°C, 95%); LTHH + MT (200 μM melatonin, 15/10°C, 95%). Results were presented as means ± SD (*n* = 3), significance values were presented as: *P < 0.05; **P < 0.01; ***P < 0.001; ****P < 0.0001; while non-significant (ns) (*P* > 0.05).

We measured the activities of antioxidant enzymes in cucumber seedlings under LT and HH conditions. Statistical analysis of the data revealed considerable differences in enzyme activities among the different treatments (Figure 3). In the LTHH treatment, enzyme activities initially increased and then declined at 6 DAT. At 4 DAT, the activities of SOD, POD, CAT, APX, MDHAR, DHAR, and GR increased by 97.67, 188.85, 496.97, 166, 401.67, 289.58, and 164.61%, respectively, in the LTHH treatment compared with the control. Then, the enzyme activities gradually decreased before peaking at 6 DAT. In the LTHH + MT treatment, the activities of SOD, POD, CAT, APX, MDHAR, DHAR, and GR were increased by 201.3, 213.92, 477.63, 205.54, 434.47, 385.52, and 224%, respectively, at 4 DAT.

### Expression of Antioxidant Enzyme-Encoding Genes

**FIGURE 4:**
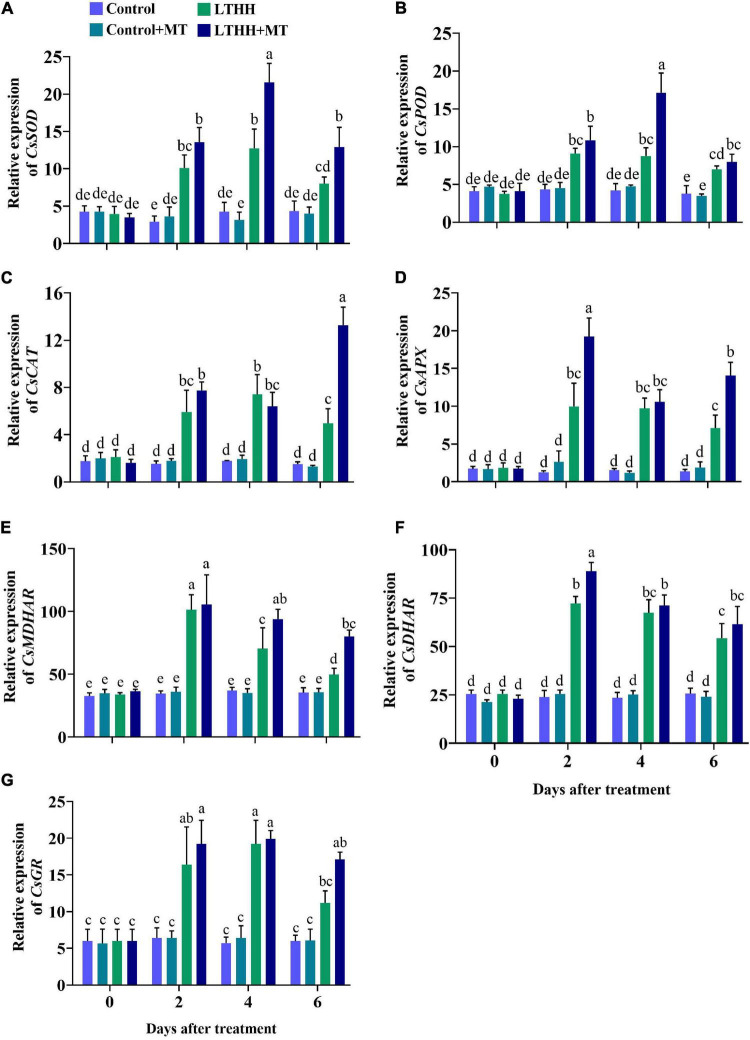
Effect of exogenous melatonin on relative expression of SOD **(A)**, POD **(B)**, CAT **(C)**, APX **(D)**, MDHAR **(E)**, DHAR **(F)**, and GR **(G)** in cucumber seedling under low-temperature and high-humidity stress. Control (25/18°C); Control + MT (200 μM melatonin, 25/18°C); LTHH (15/10°C, 95%); LTHH + MT (200 μM melatonin, 15/10°C, 95%). Same letters on the bars represent no significant differences, while different letters represent significant differences among treatments (*P* > 0.05 was non-significant). Data are means ± SD of three replicates (*n* = 3).

The expression profile of the genes involved in the antioxidant defense mechanism was studied under LT and HH conditions. In the LTHH treatment, the antioxidant enzyme-encoding genes including *CsSOD, CsPOD, CsCAT, CsAPX, CsMDHAR, CsDHAR*, and *CsGR* were significantly upregulated at 2 and 4 DAT but downregulated 6 DAT (Figure 4). Compared with the LTHH treatment, the expression of these genes was upregulated in the LTHH + MT treatment at all time points. Additionally, MT significantly upregulated the expression levels of *CsSOD, CsPOD*, and *CsGR* by 69.25, 95.33, and 3.38%, respectively, at 4 DAT, and those of *CsAPX, CsMDHAR*, and *CsDHAR* by 92.54, 4.12, 23.21%, respectively, at 2 DAT. *CsCAT* was only the gene upregulated in the LTHH + MT treatment (by 166.53%) at 6 DAT compared with the LTHH treatment (Figures 4D–F).

### Chlorophyll Content

**FIGURE 5:**
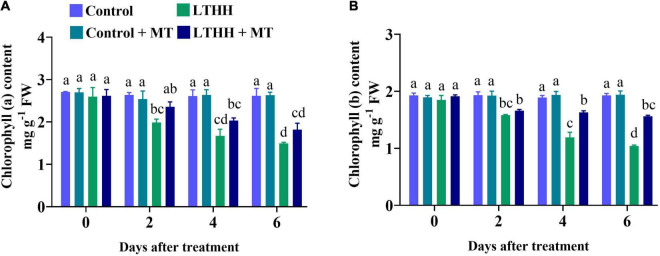
Effect of exogenous melatonin on chlorophyll *a* **(A)**, and chlorophyll *b* **(B)** content of cucumber seedling under low-temperature and high-humidity stress. Control (25/18°C); Control + MT (200 μM melatonin, 25/18°C); LTHH (15/10°C, 95%); LTHH + MT (200 μM melatonin, 15/10°C, 95%). Same letters on the bars represent no significant differences, while different letters represent significant differences among treatments (*P* > 0.05 was non-significant). Data are means ± SD of three replicates (*n* = 3).

Low temperature and HH stress can reduce photosynthesis and damage chlorophyll pigments; both these traits are interconnected with each other. The contents of chlorophyll a and chlorophyll b were significantly affected by the LTHH treatment (Figure 5). Compared with plants grown under control conditions, those treated with LTHH exhibited decreased levels of chlorophyll a and chlorophyll b at various time points, beginning at 2 DAT (Figures 5A,B). At 6 DAT, the contents of chlorophyll a and chlorophyll b were decreased by 42.90 and 46.03%, respectively, in the LTHH treatment compared with the control. However, in the LTHH + MT treatment, chlorophyll a and b contents were significantly increased by 30.86 and 19.44%, respectively, at 6 DAT (Figure 5). This suggests that MT application reduces the degradation of chlorophyll pigments in cucumber seedlings under LT and HH conditions.

### Carotenoid Content

**FIGURE 6:**
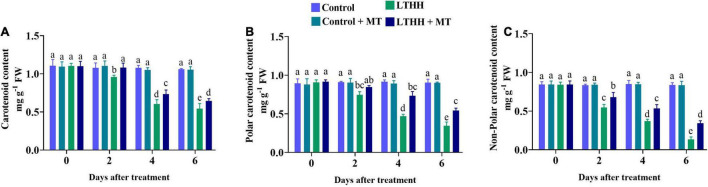
Effect of exogenous melatonin on carotenoid **(A)**, polar carotenoid **(B)**, and non-polar carotenoid **(C)** content of cucumber seedling under low-temperature and high-humidity stress. Control (25/18°C); Control ± MT (200 μM melatonin, 25/18°C); LTHH (15/10°C, 95%); LTHH + MT (200 μM melatonin, 15/10°C, 95%). Same letters on the bars represent no significant differences, while different letters represent significant differences among treatments (*P* > 0.05 was non-significant). Data are means ± SD of three replicates (*n* = 3).

Photosynthesis is extremely sensitive to LTHH stress, which can impede photosynthesis regulation by reducing plant chlorophyll and carotenoid contents. In this study, the content of carotenoid was significantly lower in the LTHH treatment at 2 DAT compared with the control and control + MT treatments (Figure 6). However, in the LTHH + MT treatment, the contents of carotenoid, polar carotenoid, and non-polar carotenoid were significantly increased at each time point (Figure 6). At 6 DAT, the carotenoid, polar carotenoid, and non-polar carotenoid contents were significantly lower in the LTHH treatment (by 48.60, 61.98, and 83.99%, respectively) than in the control treatment. Exogenous MT application to LTHH treated cucumber seedlings alleviated the reduction in their carotenoid contents, and the carotenoid, polar carotenoid, and non-polar carotenoid contents were increased by 38.84, 39.75, and 58.88%, respectively, at 6 DAT.

### Nutrient Contents

**FIGURE 7:**
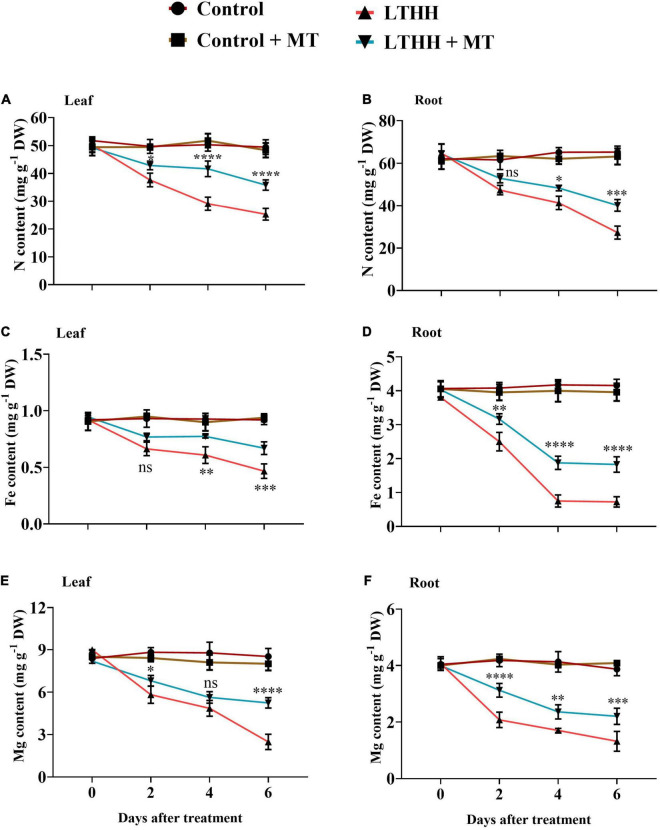
Effect of exogenous melatonin on nitrogen content **(A,B)**, iron content **(C,D)**, and magnesium content **(E,F)**, in root and leaves of cucumber seedling under low-temperature and high-humidity stress. Control (25/18°C); Control + MT (200 μM melatonin, 25/18°C); LTHH (15/10°C, 95%); LTHH + MT (200 ^μ^M melatonin, 15/10°C, 95%). Results were presented as means ± SD (*n* = 3), significance values were presented as: *P < 0.05; **P < 0.01; ***P < 0.001; ****P < 0.0001; while non-significant (ns) (*P* > 0.05).

The LTHH treatment significantly affected nutrient uptake in cucumber seedlings. Compared with the control, the LTHH treatment reduced the contents of N, Mg, and Fe in leaves at each time point; the N, Mg, and Fe concentrations were reduced in leaves by 48.85, 70.89, and 49.24%, respectively, at 6 DAT (Figures 7A,C,E). However, pretreatment with MT in the LTHH + MT treatment significantly increased the N, Mg, and Fe concentrations in leaves by 25.88, 34.54, and 27.17%, respectively, at 6 DAT. Additionally, under LTHH conditions, the root N, Mg, and Fe concentrations were decreased by 58.19, 65.90, and 82.58%, respectively, at 6 DAT compared with the control treatment; however, MT pretreatment increased these nutrients by 36.46, 46.07, and 53.84%, respectively, at 6 DAT (Figures 7B,D,F).

### MDA, Electrolyte Leakage, H_2_O_2,_ and O_2_^–^ Contents

**FIGURE 8:**
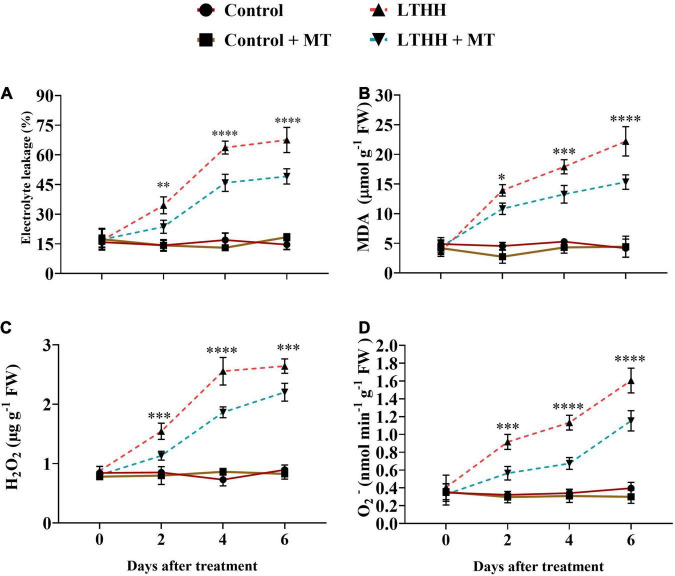
Effect of exogenous melatonin on electrolyte leakage **(A)**, malondialdehyde **(B)**, hydrogen peroxide **(C)**, and superoxide anion **(D)** content of cucumber seedling under low-temperature and high-humidity stress. Control (25/18°C); Control + MT (200 μM melatonin, 25/18°C); LTHH (15/10°C, 95%); LTHH + MT (200 μM melatonin, 15/10°C, 95%). Results were presented as means ± SD (*n* = 3), significance values were presented as: *P < 0.05; **P < 0.01; ***P < 0.001; ****P < 0.0001; while non-significant (ns) (*P* > 0.05).

We also measured MDA and EL in the leaf samples to investigate whether MT alleviates LTHH stress that induces damage in cucumber seedlings. The LTHH treatment increased EL and induced dramatic accumulations of MDA in cucumber seedlings compared with the control treatment. A dramatic increase was observed in EL and MDA content between 2 and 4 DAT, and the increase continued until 6 DAT. A significant increase in MDA content and EL (431.37 and 362.23%, respectively) was observed at 6 DAT in the LTHH treatment. However, in the LTHH + MT treatment, the MDA content and EL were decreased by 246.1 and 167.58%, respectively, at 6 DAT (Figures 8A,B).

To investigate the degree of LT- and HH-induced oxidative damage to cell membranes, we measured the H_2_O_2_ and O_2_^–^ contents. The H_2_O_2_ content showed a sharp increase at 4 DAT, followed by a slight increase at 6 DAT. However, MT pretreatment (LTHH + MT) significantly reduced this increase, especially at 2 and 4 DAT (Figure 8C). A marked increase was observed in O_2_^–^ generation rate over time under the LTHH conditions (Figure 8D), whereas the LTHH + MT treatment significantly suppressed the production of O_2_^–^ at all time points.

## Discussion

Because of its economic significance, cucumber is increasingly being cultivated in various countries including China. In recent years, high tunnels have been preferred for planting cucumber to meet the market demand. However, because of the LT in northern regions, both LT and HH pose a considerable threat to the growth and development of cucumber plants. Therefore, strategies for improving the stress tolerance potential of cucumber plants are urgently needed. Such approaches would help to increase cucumber yield and quality and ensure global food security (Ahanger et al., 2020). MT has been implicated in plant tolerance to various environmental stresses, including LT, heavy metal toxicity, high salinity, and drought (Bajwa et al., 2014; Zuo et al., 2014; Li et al., 2016; Arora and Bhatla, 2017). However, no report is available on the role of MT in reducing the stress imposed by LT and HH in cucumber. In this study, we show that MT application improves cucumber seedling growth under LT and HH stress by regulating seedling morphology and improving photosynthesis, antioxidant enzyme defense, and nutrient uptake. Consistent with our recent report (Amin et al., 2021), LT and HH obstructed cucumber seedling growth by negatively affecting the plant morphological traits and biomass accumulation, and foliar application of MT effectively alleviated these inhibitory effects of LTHH stress. These results are also in agreement with the study of Li et al. (2017), which showed that the growth and biomass of plants reflect their sensitivity or resistance to abiotic stresses. The reduction in morphological traits might be related to photosynthesis and the antioxidant defense system. LT and HH affect photosynthesis by impacting carbon (C) fixation and electron transport in thylakoids (Amin et al., 2021). LT and HH damage photosynthesis by affecting energy production. Ninety-five percent of the dry matter content of plants is derived from photosynthesis and forms the basis of crop yield (Zhang et al., 2021). The decline in photosynthetic efficiency is the main reason for the reduction in plant biomass under LT and HH conditions (Amin et al., 2021). Photosynthesis is a complex process that involves numerous biological pathways. Environmental stressors, such as cold, salinity, and drought, impair photosynthetic C metabolism in plants, resulting in stunted plants and reduced yield (Hutchison et al., 2000; Ding et al., 2017b; Wang et al., 2020). In the photosystem reaction center, an extreme stress environment causes severe photoinhibition. When the dissipation mechanism fails, plants utilize excess energy, which damages the photosystem reaction center (Jahan et al., 2021). In this study, we evaluated the parameters of PSI and PSII to unveil the damaging effects of LT and HH on photosynthesis, PSII activity, and the role of MT in plant defense (Figure 1). MT application triggers many genes and enzymes associated with C and N metabolism, which in turn improves photosynthesis and consequently promotes plant growth and development under abiotic stress conditions (Ren et al., 2021). We found that Pn, Gs, and E, were markedly reduced by LTHH stress, which is consistent with previous studies (Ding et al., 2017a,^2018^) in which exogenous application of MT enhanced photosynthesis under cold stress. The resumption of photosynthesis and plant growth is important for survival under stress conditions (Wang et al., 2020). In this study, MT application facilitated the recovery of photosynthesis after LTHH-treated cucumber seedlings were reverted to normal growth conditions. Moreover, evidence related to the regulatory mechanisms underlying the beneficial effect of MT on photosynthesis under LT and HH conditions, particularly in chilling-sensitive crop species, is limited. Previous studies showed that the decline in photosynthesis during chilling stress is mainly caused by a reduction in the activity of photosynthetic enzymes, PSII, and PSI (Bi et al., 2015). Consistently, we found that LT and HH reduced the values of Fv/Fm, qP, and ETR (Figure 2); however, PSII activity was higher in cucumber seedlings subjected to the foliar application of MT than in those subjected to the LTHH conditions. Investigation of the PSII reaction center and its donor and acceptor sites is considered important for PSII activity in plants, especially under abiotic stress conditions (Li et al., 2005). Chlorophyll is essential for harvesting light during photosynthesis. Both LT and HH severely retard or inhibit the synthesis of chlorophyll, which directly impacts plant growth and yield (Zhang et al., 2021). Cold stress degrades photosynthetic pigments by upregulating chlorophyllase activity and downregulating enzymes involved in chlorophyll biosynthesis, thus directly affecting photosynthesis and chlorophyll fluorescence (Jahan et al., 2021). Chlorophyll is essential for photosynthesis and is highly sensitive to cold stress (Amin et al., 2021), which disrupts all key mechanisms of photosynthesis. In the current study, we verified that MT pretreatment reduces LT and HH stress-induced chlorophyll degradation and carotenoid biosynthesis, suggesting that MT can effectively stimulate chlorophyll synthesis in stressful environments.

Low temperature causes oxidative stress, which affects cell membrane function. Lipid peroxidation and EL are the primary indicators of cellular damage. In cucumber plants exposed to LTHH stress conditions, a dramatic increase in MDA and EL was detected, suggesting severe lipid peroxidation and plasma membrane damage. MT application effectively alleviated the LTHH stress-induced damage in cucumber plants (Figures 8A,B), suggesting that MT rescues cucumber seedlings from LTHH-induced oxidative stress. Our results are consistent with previous studies, in which exogenous MT application increased the LT tolerance of cucumber (Zhao et al., 2016), watermelon (Li et al., 2016), and Bermudagrass (Fan et al., 2015) by reducing the MDA content and EL. A reduction in MDA content and EL in MT-treated plants indicate reduced oxidative damage to cell membranes. We assume that ROS concentration in MT treated seedlings was lower than that in untreated seedlings under LTHH stress conditions because lower MDA content and EL represent the alleviation of oxidative damage to cell membranes. Several studies have shown that ROS accumulation is directly related to lipid peroxidation, cell integrity, and cell death (Zhao et al., 2020). The degree of oxidative injury to the cell membrane due to LT and HH was investigated by measuring the ROS content. H_2_O_2_ is a crucial ROS in plants and is generated under stress conditions. Accumulation of ROS hastens cell membrane lipoylation and leads to the production of MDA, thereby increasing plant membrane permeability and compromising membrane structural integrity. The application of MT reduced ROS accumulation in the present study. MT acts as an antioxidant in plants under harsh environmental conditions (Wang et al., 2020). In our experiment, cucumber seedlings exposed to LTHH stress exhibited oxidative stress compared with those grown under control conditions; however, the foliar spray of MT reduced the production of ROS (Figures 8C,D). MT is considered a free radical scavenger and an antioxidant that can eliminate H_2_O_2_ for maintaining cellular homeostasis (Tan et al., 2007); this phenomenon has been reported in apple (Wang et al., 2012) and cucumber (Marta et al., 2016).

Reactive oxygen species are directly or indirectly linked with the antioxidant defense system and affect antioxidant enzymes. Many researchers have argued that exogenous MT application strengthens the endogenous antioxidant defense mechanism against climate change (Kingston-Smith et al., 1997; Cano et al., 2003; Wang et al., 2020). To cope with oxidative stress induced by adverse environmental conditions, higher plants have developed an antioxidant defense system that includes antioxidant enzymes (Zhang et al., 2014). SOD, POD, CAT, and APX are four crucial enzymes in the plant defense system. SOD can reduce O_2_^–^ to produce H_2_O_2_, which is subsequently completely decomposed by POD and CAT, producing H_2_O and O_2_ and attenuating membrane damage (Jun et al., 2008). Furthermore, except for the scavenging antioxidant enzymes mentioned above, a number of other enzymes detected in various subcellular compartments also play a role in maintaining redox homeostasis by directly scavenging specific ROS. Such antioxidant enzymes include DHAR, MDHAR, and GR. These enzymes work together to perform synergistic functions *via* the ascorbate–glutathione cycle, which operates in plant cell organelles (Gupta et al., 2018). MT boosts the plant antioxidant defense mechanism against abiotic stresses by enhancing antioxidant activity and the expression of corresponding genes (Sun et al., 2019). In a previous study, foliar application of MT enhanced the antioxidant enzyme defense mechanism and reduced the accumulation of ROS in wheat seedlings exposed to cold stress (Li et al., 2018). Consistently, in the current study, we found that exogenous application of MT dramatically elevated the activity of antioxidant enzymes and relative messenger RNA (mRNA) abundance at all time points, thus enabling plants to eliminate excess ROS (Figures 3, 4). Exogenous application of MT treatment significantly increased the expression levels of *CsSOD*, *CsPOD*, *CsCAT*, *CsAPX*, *CsMDHAR*, *CsDHAR*, and *CsGR*, and decreased the oxidative damage (Figure 4). Similar trends were reported in watermelon and in cucumber seedlings grown under chilling and drought stress conditions (Liu et al., 2015; Zhang et al., 2021). We speculate that MT exerts a positive influence on the activity of antioxidant enzymes. Interestingly, MT pretreatment enhanced the activities of these enzymes, thus maintaining low H_2_O_2_ content during the LTHH stress. This was also confirmed in trials with apple and watermelon plants, where MT directly scavenged H_2_O_2_ and boosted the activities of antioxidant enzymes to detoxify H_2_O_2_ in plants treated with cold stress (Li et al., 2017) or drought stress (Wang et al., 2013). These arguments suggest that MT plays a crucial role in plant stress mitigation.

Nutrient uptake and compartmentalization are important not only for normal plant growth and development but also for maintaining plant health under unfavorable environmental conditions that disrupt ion homeostasis (Zhang et al., 2017). LT stress has been shown to inhibit the uptake and transport of macro- and micro-nutrients in cucumbers (Anwar et al., 2018). Among the various plant nutrients, N, Mg, and Fe are responsible for chlorophyll biosynthesis as well as for other physicochemical reactions associated with plant growth and development. A deficiency of these elements can decrease photosynthetic activity (Zhang et al., 2017). In this study, we noted that the LTHH treatment decreased the N, Mg, and Fe contents of cucumber seedlings in underground and aboveground plant parts (roots and leaves). However, the MT pretreatment of stressed plants promoted the uptake of N, Mg, and Fe and their translocation from roots and leaves. The enhanced concentrations of N, Mg, and Fe in MT-treated plants suggest that MT has a positive influence on plant growth and development (Anwar et al., 2018). MT defends against chlorophyll degradation and increases plant growth under stressful environments, perhaps because it upregulates the accumulation of N, Mg, and Fe under such conditions (Ahammed et al., 2020).

## Conclusion

The findings of this study revealed that MT mitigates the damaging effects of LT and HH on cucumber seedlings. LT and HH affected the morphological attributes of cucumber seedlings, inhibited photosynthesis, and affected nutrient uptake by increasing ROS contents, lipid peroxidation, and electrolyte leakage in cucumber seedlings. However, MT rescued the growth of cucumber seedlings under LT and HH conditions by improving morphometric traits, increasing antioxidant enzyme activities and transcript levels, regulating PSI and PSII, enhancing nutrient uptake, and alleviating oxidative damage. Our findings demonstrate that the foliar application of MT reestablishes cucumber seedling growth under LT and HH stress conditions. Our work provides a case study that exogenous application of melatonin may serve as a practical approach to improve LT and HH tolerance in cucumber production. Future studies should include the integrity of chloroplast structure and molecular characterization of cucumber to improve understanding of the underlying mechanisms of LTHH stress and melatonin response.

## Data Availability Statement

The raw data supporting the conclusions of this article will be made available by the authors, without undue reservation.

## Author Contributions

BA, ZC, HM, and SL: conceptualization. BA, MJA, HFA, AM, and KRH: methodology. BA and HFA: software. BA, AM, KRH, and MA: validation. BA: formal analysis, investigation, and writing—original draft preparation. MJA and ZC: writing—review and editing. ZC: resources, supervision, project administration, and funding acquisition. All authors contributed to the article and approved the submitted version.

## Conflict of Interest

The authors declare that the research was conducted in the absence of any commercial or financial relationships that could be construed as a potential conflict of interest.

## Publisher’s Note

All claims expressed in this article are solely those of the authors and do not necessarily represent those of their affiliated organizations, or those of the publisher, the editors and the reviewers. Any product that may be evaluated in this article, or claim that may be made by its manufacturer, is not guaranteed or endorsed by the publisher.

## Footnotes

1
www.graphpad.com
