# Supplementary material for: Melatonin Rescues Photosynthesis and Triggers Antioxidant Defense Response in Cucumis sativus Plants Challenged by Low Temperature and High Humidity
Source: Front Plant Sci. 2022 Apr 27;13:855900. doi: 10.3389/fpls.2022.855900 (PMC9094117; doi:10.3389/fpls.2022.855900)
Supplement: Supplementary file 1 [file Table_1.DOCX]

**Supplementary Table 1.** Primers used in this study

| **Gene ID** | **Gene name** | **Primer sequence (5’-3’)** |
| --- | --- | --- |
| *CsaV3_4G004420* | *CsSOD* | L: TTGACCATCCACGTCCATCA  R: AGTCTGCTGCTTCCCATGAT |
| *CsaV3_4G023590* | *CsPOD* | L: TCGTTGCTCTATCAGGTGCA  R: ATGTGCCTGACCCTGATTGA |
| *CsaV3_6G031490* | *CsCAT* | L: TCCCGAAAGCCTTCTGTCTT  R: GAGTGGCGTGACTGTGATTC |
| *CsaV3_6G034310* | *CsAPX* | L: TGCCGCCAAATATTCGTACG  R: TGGCCAAAACAGCAATCACA |
| *CsaV3_4G002100* | *CsGR* | L: GGATTTACTGTGGCTGTGAAAA  R: CTGCTGCATCTTTAGCCTCA |
| *CsaV3_5G027310* | *CsMDHAR* | L: TTTGGGGCATATTGGGTAAA  R: ATAACTCAGTCCCCGGCTTT |
| *CsaV3_5G006680* | *CsDHAR* | L: AAGGAAAAGTGCCAGTGGTG  R: AAGCGCCTTCAACTCTTCAA |
| *Actin* |  | R: CCACGAAACTACTTACAACTCCATC  L: GGGCTGTGATTTCCTTGCTC |
